# Management of industrial high-pressure fluid injection injuries (IHPFII): the Water Jetting Association (WJA) experience with water driven injuries

**DOI:** 10.1007/s00068-019-01106-4

**Published:** 2019-03-20

**Authors:** Sancho Rodríguez-Villar, Robert Charles Kennedy, Martino Dall’Antonia, Carlos Pilasi Menichetti

**Affiliations:** 1 King’s College Hospital NHS Trust Foundation, Denmark Hill, Brixton, London, SE5 9RS UK; 2 The Water Jetting Association, Thames Innovation Centre, 2 Veridion Way Erith, Kent, DA18 4AL UK; 3 Lewisham and Greenwich NHS Trust Foundation, Stadium Rd, Woolwich, London, SE18 4QH UK

**Keywords:** Industrial high-pressure fluid injuries, High-pressure fluid injection, Water jetting, Hydrostatic injuries, Ultra-high pressure, High-pressure water jet injury, Water jet injury

## Abstract

**Background:**

Industrial high-pressure fluid injection injuries (IHPFII) are largely occupational in nature, where these injuries are most often sustained by male manual workers. Such traumatic injuries are largely sustained with water, grease, paint, gasoline or paint thinner. IHPFII are extremely serious injuries with life and limb-threatening potential carrying the risk of life-long disability.

**Methods:**

We reviewed the Water Jetting Association© adverse incident database of advisory alerts detailing cases from around the world that have been brought to the association’s attention and the English-language literature on high-pressure hydrostatic injuries from 1937 to 2018.

**Results:**

Accidents involving high-pressure water jets in the industry are uncommon. The clinical impact in all of the cases reviewed and the effects of water jet impacts range from instant fatalities at scene to loss of limb function and amputation. The majority of observed fatalities are due to major hemorrhage (exsanguination) secondary to the direct dissection of great vessels or high-energy blunt soft tissue injury and traumatic brain injury.

**Conclusions:**

As with any other trauma, IHPWJI commonly result in amputation or death. Nonetheless, a lack of comprehension of the potential severity of injuries and range of infective complications appears to be largely due to the apparent benignity of the initial presentation of the wound. This in turn leads to delays (both avoidable and unavoidable) in the transfer to appropriate medical facilities and definitive care. There is an identifiable need for education (including for health care providers across multiple levels), training and the availability of personal trauma kits for the timely and effective management of IHPWJI from the initial jet impact on the scene, as well as a need for an established referral system.

## Background

**Fig. 1:**
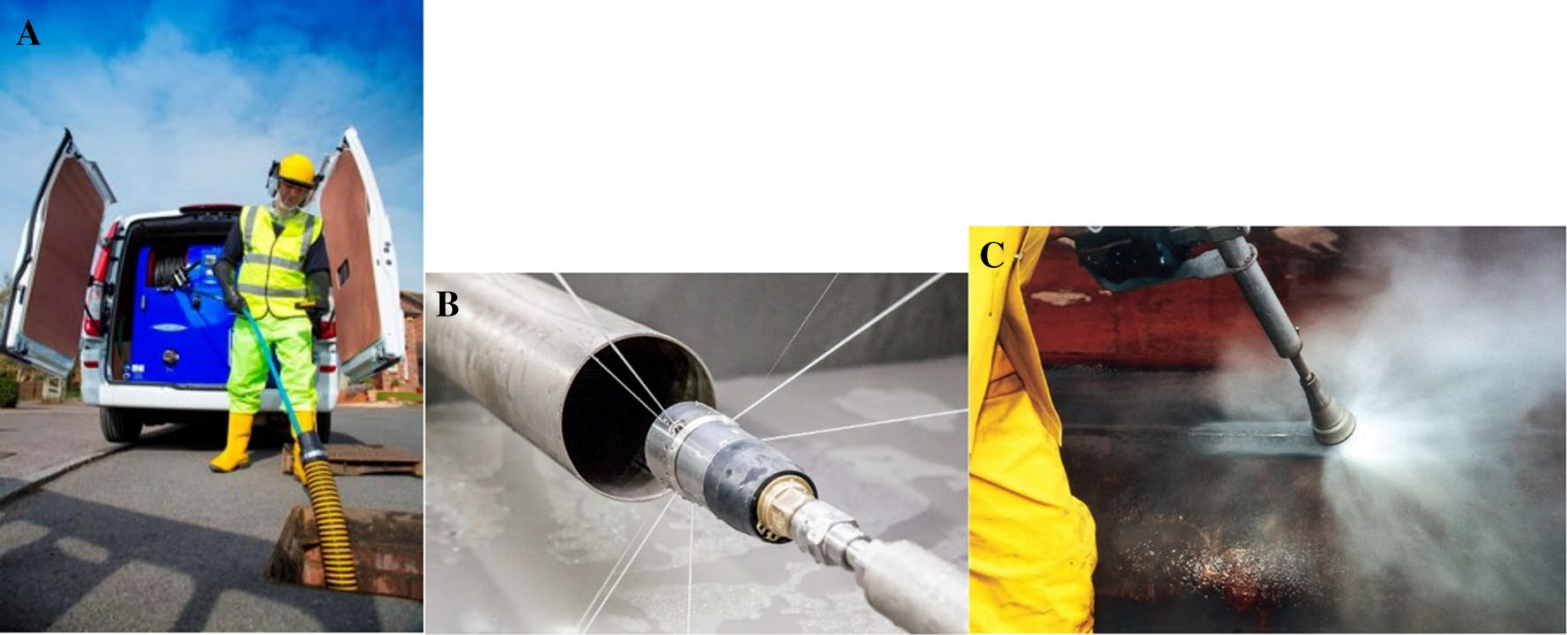
**a** An operator with a mobile unit working in a drain cleaning setting, across an estimated working pressure up to 206 bar (= 204 atm = 3.000 psi). The unit would be (on average) operating at 12–15 gallons (US) per minute (= 45–55 L/min). **b** An example of a spinning/rotary nozzle. Generally used for the full circumference cleaning of pipes and drains, particularly effective for removing fats and grease and the de-scaling of pipes and drains. The drain cleaning nozzles tend to use round (pencil jets) which in terms of an accident will definitely cause more damage to the body than a fan jet. **c** Ultra-high pressure (UHP) operations begin at a level over 1.700 bar (= 1.677,77 atm = 24.656,42 psi). At UHP, the jet velocity is in excess of 2.400 km/h, when a pencil jet is used this jet is the most concentrated and is subsequently capable of creating the most severe operator injuries

The Water Jetting Association© (WJA) encompasses more than 200 companies that operate around the world and perform an array of industrial services. The association was formed over 35 years ago by a small group of like-minded contractors, manufacturers and machine/equipment hirers who were all committed to raising safety standards within the then emerging high-pressure water jetting industry [1, 2]. The WJA has always been concerned with safety and training within the water jetting industry for such activities as pressure washing, high-pressure/ultra-high-pressure operations and drain and sewer cleaning that spans the five continents (Fig. 1).

A hydraulic injection can be defined as the puncturing of the epidermis by a jet of a fluid under pressure. The pressure required to pierce the full skin is approximate of 39, 5 atm (= 40 bar = 580 psi). Pressures currently used for ultra-high-pressure water jetting can exceed 2.467 atm (= 2.500 bar = 36.259 psi), a jet velocity up to 2.500 km/h (= 1550 mph) and can deliver 60 gallons (US)/min (3, 8 l/s) [1, 2].

Hydraulic injection injury occurs when a jet of fluid under pressure penetrates the skin of an individual, often from a momentary human error, such as tripping or falling, while operating equipment. Equipment failures due to fatigue cracking in high-pressure couplings and hose lines, pinhole-sized failures in hydraulic hoses, seal failure and bulk material cracking are secondary causes [1, 2].

Although high-pressure water jet injection injuries to soft tissues were first described in the 1930s, these injuries have rarely been reported, especially considering the current widespread use of the equipment [3].

The aim of this review is to raise the level of awareness of the potential severity of injuries of this type, irrespective of the constituent fluid injected. The paper seeks to improve the overall prognosis by adding to the extant knowledge base that our experience (and learning points) gained from the management of previous accidents through the water jetting industry in recent years, to encourage the companies to report the incidents and to present a clear, simple and updated approach to the management pathway.

## Methods

We reviewed and followed up the Water Jetting Association© (WJA) adverse incident database of advisory alerts for the last 5 years detailing cases from around the world that have been brought to the association’s attention and the English-language literature on high-pressure hydrostatic injuries from 1937 to 2018.

## Results

A total of 42 clinical cases were included. Only 26 reported cases of high-pressure water jet injury related to industry could be found in the literature. 16 adverse incidents were included from over 200 companies contacted through the WJA from around the world in the last 5 years, from those: only five companies replied to our invitation to study the circumstances of the accident. We were able to contact only with nine patients and to be involved directly with two cases occurring during this study (these two patients did not reply to our follow-up invitation for unknown reasons). From all cases, the upper limb (hand and forearm) were the more frequent site of injury, found in 21 cases. Other injury sites were the groin and lower limb (sixteen); abdomen (seven); neck (three); chest (two); head (two); eyes (three) and fatality (four).

## Mechanism of injury

Three significant factors that play important roles in prognosis have been reported and confirmed in our study for these injuries. Firstly, the physical injury can cause damage to local soft tissues resulting in the water (or irritant material) traveling proximally along visceral planes, nerves or tendon sheaths, leading to vascular compression and local necrosis. Secondly, the chemical properties of the injected material can cause compressive vascular injuries with increased edema and inflammation. Thirdly, injuries can be contaminated with virulent organisms or foreign material, thereby leading to unusual infections.

### Physical mechanism

**Fig. 2:**
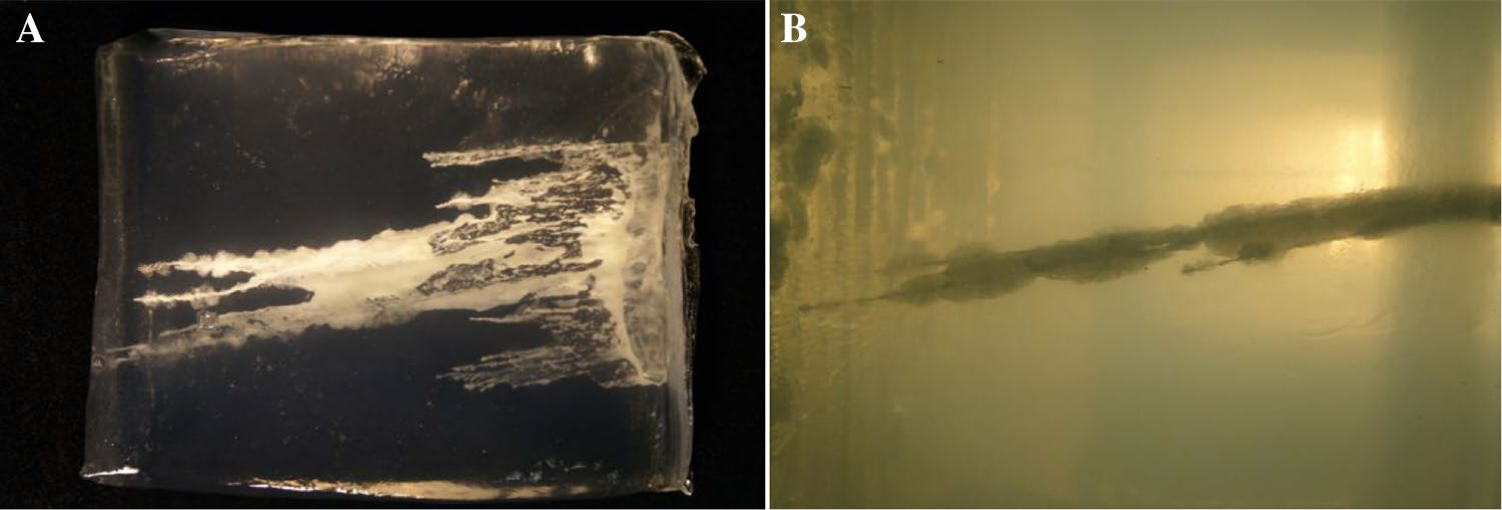
Study of water jet impact on ballistic gel blocks. **a** Clearly shows disruption of the tissue simulant and a significant quantity of fluid retained in the wound. Prepared cross section of ballistic gelatine wound path. **b** The remains of the bubble-like cavities created during the propagation of the wound path

Previous research, most recently that conducted by McDonald [4] using ballistic gelatin blocks was able to capture high-speed video of a typical wound path created by injury through hydraulic injection. Post-test analysis of the high-speed video showed that the jet of fluid easily penetrated the tissue simulant, the pattern of tissue damage can be similar to that of a high-velocity missile gunshot wound (Fig. 2).

The primary factors that contribute to achieving hydraulic injection are the physical dimensions of the water jet, the pressure of the water jet itself and the operator’s proximity to it.

**Fig. 3:**
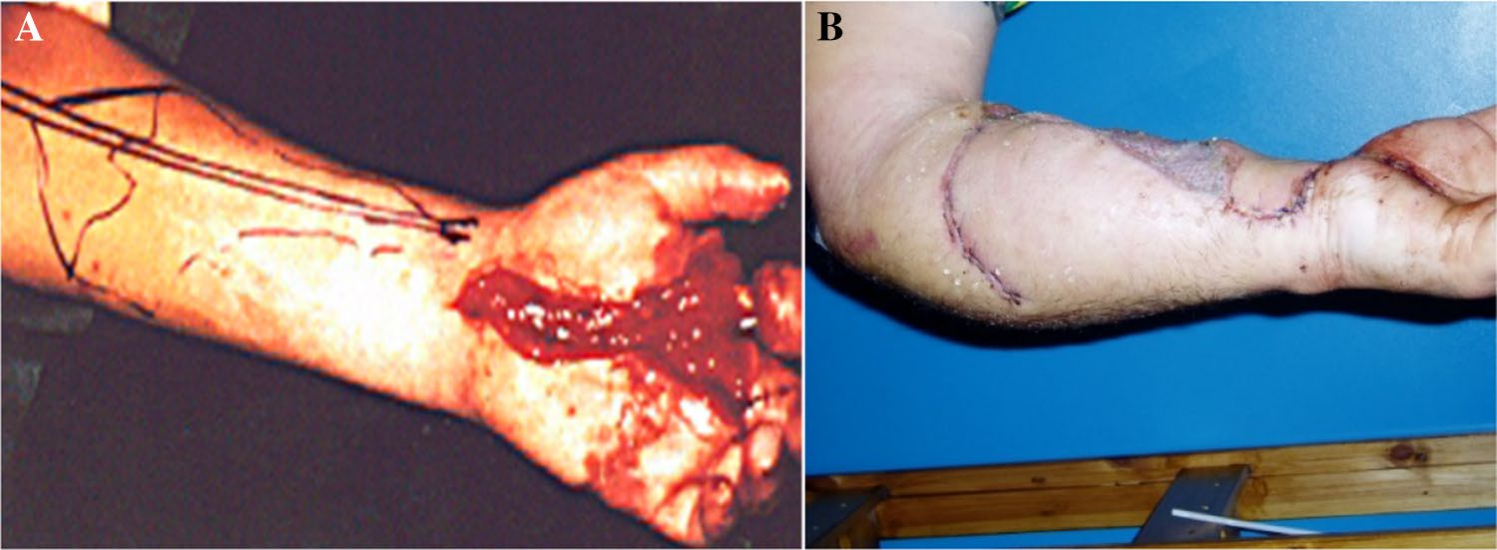
**a** The patient was admitted to hospital after an accident cleaning concrete, he underwent an urgent decompression and extensive debridement, the wound left open and had three more consecutive and increasingly extensive surgical explorations at incident plus 1 day and incident plus 4 days. **b** Forearm and hand after almost 1 year elapsed time from an accident and after two plastic surgery reconstructions requiring an abdominal flap. **c** External tank cleaning operations

**Fig. 4:**
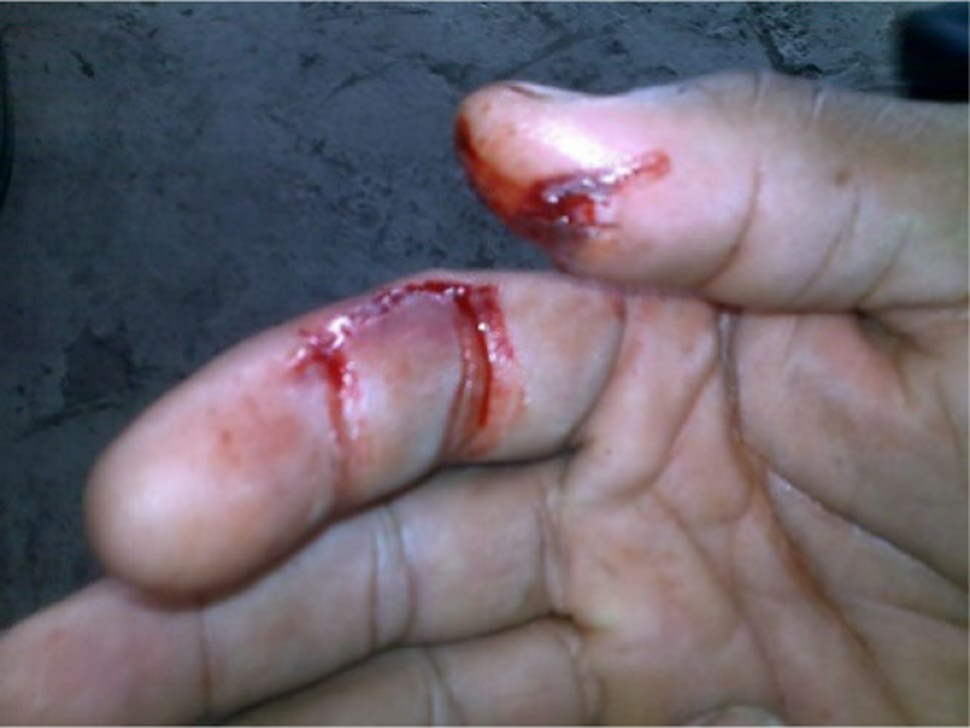
A 52-years-old male operator who sustained a high-pressure water jet injury on his 4th and 5th right fingers (a proximal phalanx of the index and distal phalanx of thumb). The patient presented in A&E (ED) after 9 h post-injury when the pain started and he lost the sensation and functionality. An extensive debridement was performed including decompression of carpal tunnel

When a high-pressure fluid injection injury occurs, the kinetic energy absorbed by the tissues is substantial. The material is often driven from the fingertip to the palm, which is usually seen in the non-dominant hand of young men who commonly work in the industrial sector (Figs. 3, 4).

The common encounter is of a very small entrance wound and lack of an exit wound are not indicative of the extensive disruption of deeper tissues that can result from dispersion of kinetic energy penetration of the skin by water (Fig. 4).

**Fig. 5:**
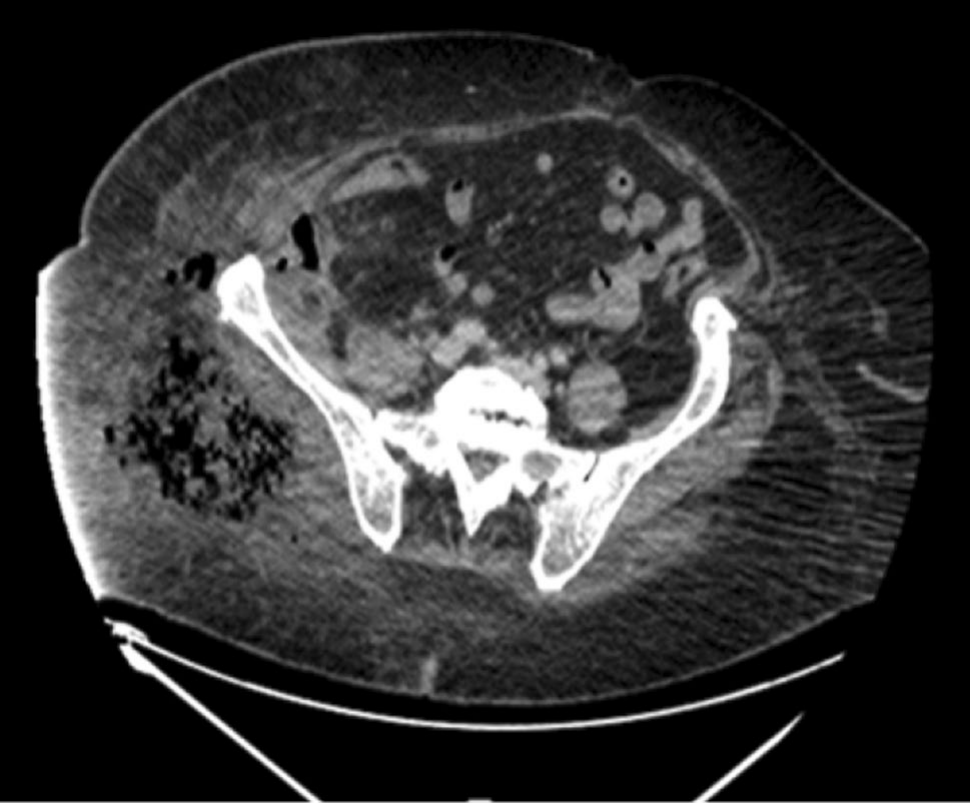
A 43-years-old male operator who sustained a high-pressure water jet injury in the right thigh, where the picture shows a CT-scan at the level of thigh and pelvis. There is a large right gluteal collection of gas bubbles associated with inflammation and edema extending to the lateral abdominal wall subcutaneous tissues. A smaller complex air-fluid collection is also noted deep to the lateral lower abdominal wall and in the right iliacus muscle

High-pressure water jet injuries may result in the infiltration of water and air into the tissue planes. The resulting subcutaneous emphysema can be an indication of the extensive internal damage. A classic radiographic appearance of diffuse subcutaneous air may be identified by CT-scan imaging (Fig. 5).

High-pressure water jet injuries present with several unique features. The external manifestations of the injury are unreliable for predicting the extent of internal damage [5–8]. Certain cases result in the development of intra-compartmental pressure in compartments of the limbs (which are bonded by bone and fascia and contain muscle, nerves, and vessels) (Fig. 5). Wounds of the abdominal wall may involve intraperitoneal injuries. The amount of energy transferred and the degree of muscle damage with subsequent rhabdomyolysis can put the kidney perfusion at risk. In such cases, hospital admission must be considered.

### Nature of the fluid

The nature of the fluid responsible for the injury is also important, as its specific gravity and mass will also have a degree of influence on both the velocity and kinetic energy delivered. Paint, gasoline, grease and fuel oil are common agents used in high-pressure or hydraulic spray guns (or found as contaminants). Non-isotonic and tissue-toxic substances can exacerbate compressive vascular injuries with increased edema and inflammation. A meta-analysis of HPII concluded that the type of injected material was the most important factor affecting outcomes. Water injection injuries do not result in the same degree of secondary tissue damage and toxicity. The authors found that 4 of 5 (80%) patients injected with paint thinner or turpentine require eventual amputation, whereas only 9 of 40 (22.5%) patients injected with grease, a considerably less caustic agent, required amputation [4, 7, 9]. Paint and paint solvents appear to be the most irritating to tissues, with a 60–80% amputation rate being reported.

### Infection (microbiology)

Due to the high volumes of water needed by HPWJ equipment, water is often obtained from reservoirs, rivers or other environmental fresh or salt water sources where the high bacterial and fungal load can be found. Alternatively, the water used in such jetting devices usually is stored in a multitude of container types in the vehicles within which the water jetting equipment is transported, and can often be found contaminated with microaerophilic bacteria from sewage and industrial waste. It is not uncommon for operators to siphon water from a nearby pond, lake, open water supply or river for use in the water closed system of a high-pressure jetting unit via some type of water storage setup or even in direct supply via some form of in system particle filtration.

The normal flora and fauna found in proximity to a lake water supply (should any such be employed by an operator) can result in many viral, protozoal, parasitic, and bacterial pathogens being present in such a supply to a high-pressure water jetting system.

In the initial period after an injury *Staphylococcus aureus*, beta-hemolytic streptococci and *Clostridium* spp are a common cause of skin and soft tissue infection in trauma patients.

The range of pathogens capable of causing soft tissue infections after HPWJI is complicated by the presence of salt water and fresh water pathogens.

*Vibrio vulnificus* is associated with exposure to saltwater and brackish waters and can present with infections ranging from wound infection with fulminant cellulitis to myositis and necrotizing fasciitis. After hurricane Katrina, an increase in number of skin and soft tissue Vibrio was reported [10].

Freshwater exposure is often associated with *Aeromonas* spp. infections, which can cause wound infection and rapidly progress to necrotizing fasciitis. After the 2004 Asian Tsunami, *Aeromonas* spp. were the most commonly isolated organism from patients admitted in Thailand, after the Tsunami wave displaced water from inland freshwater reservoirs [10].

Soldiers with severe blast and bullet injuries repatriated in the UK from the Middle East and Asian war theaters were treated at the Queen Elizabeth Hospital in Birmingham. Infections from blast injuries sustained in the “green zone” of Afghanistan were frequent, with *Pseudomonas* spp. and *Aeromonas* spp. common pathogens (Martin Gill, personal communication).

It is essential that necrotic and infected tissue samples are collected and processed in a microbiology laboratory both for microbial culture and sensitivities and for fungal microscopy and culture.

Case reports from HPWJI often showed multiple pathogens isolated from tissue debridement, including *Clostridium* spp. and other anaerobic bacteria as well as *Escherichia coli* and *Stenotrophomonas maltophilia*, some pathogens are isolated from specific geographical areas [11]. For example, *Burkholderia pseudomallei* causes disease mainly in Southeast Asia and Australia’s Northern Territory [10]. *Chromobacterium violaceum* can cause cellulitis, abscesses and severe sepsis after freshwater exposure in tropical or subtropical areas [10].

*Leptospira* spp. are spirochetae which can be acquired after mucosal or broken skin exposure to contaminated water and which can cause a range of different presentations, from the classical Weil’s disease to leptospirosis with pulmonary hemorrhage. Leptospira does not cause wound infections, but an increase in cases of leptospirosis has been associated with floods, so the index of suspicion should be low.

Extensive repeated surgical debridement combined with antimicrobial prophylaxis to cover freshwater pathogens was employed [12].

#### Fungal infections

Fungi are present in untreated effluent and stagnant water, and can also be isolated in significant quantities from water distribution systems and in treated tap water [13]. Accumulation of fungi in water storage facilities has been described [14].

Fungi are a high prevalence component of untreated effluent water and stagnant water, and can also be isolated in significant quantities from water distribution systems and in treated tap water. A case report has shown filamentous fungi (*Fusarium* and *Acremonium* spp.) causing wound infection after IHPWJ injuries [11].

A recent case series and review of the literature identified *Mucorales, Aspergillus, Fusarium* and *Scedosporium* spp., alone or in combination, as the most common filamentous fungi capable of causing deep wound and soft tissue infections following major trauma with significant soil tissue contamination [15].

They are ubiquitous in nature and can be found on decaying vegetation and in the soil, where they produce a large number of spores and can cause disease alone or in combination with other filamentous fungi [16].

Tissue necrosis, due to the vascular invasion with consequent ischemia, was the main sign suggestive of invasive fungal infection. Prolonged antifungal therapy combined with aggressive debridement is required. Experience from treating military personnel with blast injury advocates the use of direct microscopy of debrided tissue, which can be able to demonstrate the presence of fungal structures.

#### Diagnosis of fungal infection

Post-traumatic IFI criteria used in military cohorts have been published. Standardized diagnostic criteria [16, 17] and a high index of suspicion contribute to early diagnosis and reduced mortality in the military compared to civilian injuries.

#### Antibiotic and antifungal prophylaxis and treatment

Due to the narrow entry point and lack of awareness of the potential complications, it is not uncommon for patients with IHPWJI to delay seeking medical attention. Typically, the patient will have to go to the theater for multiple debridements.

**Fig. 6:**
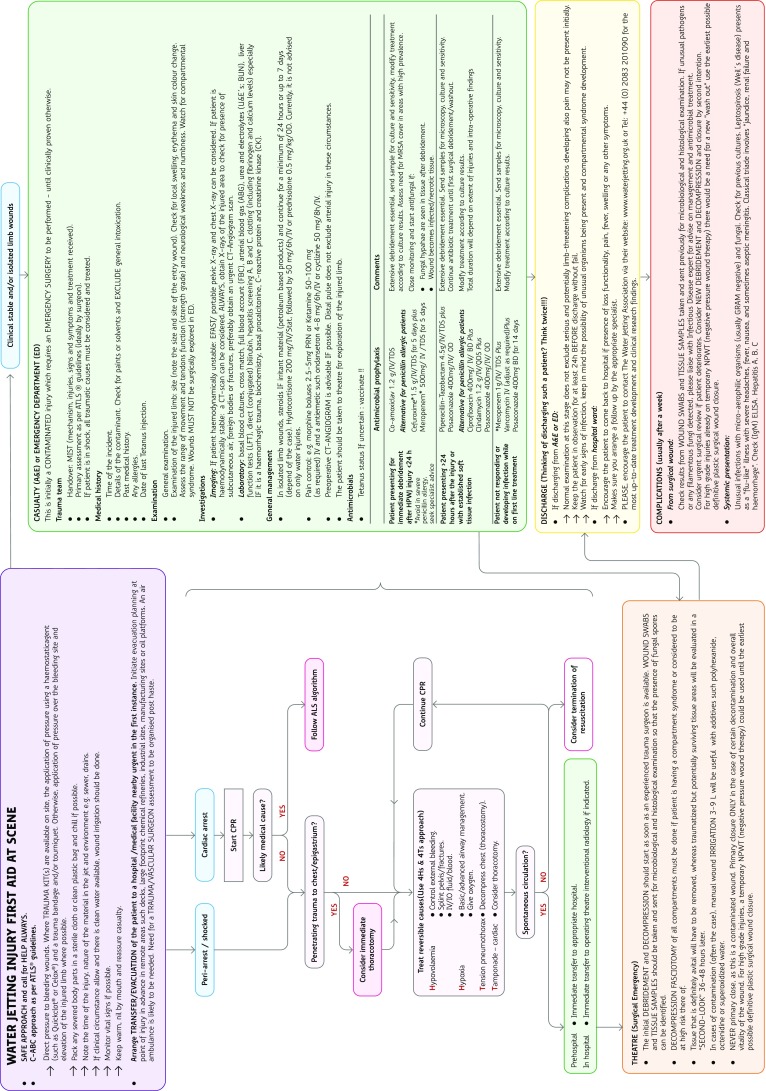
The WJA guidelines on the management of IHPFII

Empirical IHPWJI antibiotic and antifungal prophylaxis and treatment options are outlined in the management algorithm (Fig. 6), and draw on the lessons from similar clinical scenarios.

Knowledge of the environment in which IHPWJ has occurred can help to guide empirical therapy as well as the microbiology laboratory identification of suspected pathogens.

Antibiotic prophylaxis has been recommended after flooding disasters in patients presenting with high-risk wounds such as puncture-type bite wounds, crush wounds, wounds involving bone, tendon or joints, wounds with delayed treatment, and in immunocompromised patients [10].

Soldiers with blast injuries exposed to stagnant or irrigation water included are routinely offered antibiotic and antifungal prophylaxis. Drawing on experience from civilian trauma lesions and military combat wounds, antibiotic treatment is often administered for 7 days after debridement [18].

Although Zygomycete infection is usually present after a week to 10 days post-injury in military casualties with significant organic matter exposure, we suggest pre-emptive antifungal treatment in patients with delayed presentation and debridement, as high load infective material and fungal spores are likely to have been present following IHPWJ.

When fungal infection is confirmed, combination treatment with two antifungals is often required, together with prolonged treatment. Fungal isolates will have to be referred to a specialist laboratory for speciation and anti-fungal sensitivity testing. Early liaison with an infectious disease expert is essential.

#### Tetanus prophylaxis

All HPWJI patients should have their tetanus immunization status checked and managed according to the current advice on the management of patients with tetanus-prone wounds. Please refer to Chap. 30 of the ‘Green Book’ [19].

## Discussion

Due to ethical considerations, no randomized controlled trials exist to guide the management of these injuries. All data obtained in the past has been derived from isolated case studies and case series, most of which were limited to limb injuries. Very little has been published until recently concerning the causes of the fatalities and major trauma involving these injuries.

After reviewing all incidents, we have noted that there are usually three main scenarios that occur in the following order: firstly and most commonly, is an isolated limb injury (hand or foot) with no other injuries associated and the patient is clinically stable; secondly, a “Code Red” (bleeding) trauma is usually secondary to the dissection of a great vessel, commonly the femoral artery, and soft tissue blunt trauma due to a high-energy impact; and third, a severe traumatic brain injury or decapitation (sadly, most of these patients are deceased at the scene). Blunt trauma injuries are usually a result of impaction of a nozzle or lance commonly if a high-pressure hose failure (hose split) occurs. This usually causes the steel lance nozzle to “whip” out of the contractor’s hands and then “whip back” to strike across the head.

Due to the amount of energy transferred, these injuries initially must be treated as any other severe major trauma following the C-ABCD (circulation, airway and cervical spine control, breathing, circulation, and disability) approach from the ATLS^®^ (advanced trauma life support protocol) [20]. After the initial assessment, help should be sought as soon as possible, and transfer to a trauma center is always a must. Evacuation in a helicopter or plane to a trauma center with surgical facilities must be considered in the early stages.

If the patient is stable, the first image should be a contrast CT if available, and if the patient is sufficiently stable, an MRI may be considered, as well (Fig. 5).

Diagnostic adjuncts are very important, in a stable patient with an isolated limb injury, we strongly advise obtaining an MRI (if facilities are available) to study the interstitial tissue and planes and/or a contrast CT-scan if a vascular lesion is suspected.

On average, for more localized wounds, such as those of the hands or arms, patients wait several hours before seeking medical attention and, from our experience, the injury can take from 9 to 52 h to become symptomatic.

High-pressure water jet injuries should always, without exception, be considered contaminated wounds and treated as surgical emergencies. A high index of suspicion of associated internal injuries and aggressive surgical intervention is required. In a review of 435 cases, Hogan et al. [7] found that the amputation rate is proportional to the time taken to undertake surgical debridement. The amputation rate for injection injuries averaged 38% compared to an amputation rate of 58% when surgical debridement was delayed more than 6 h and averaged a devastating 88% when debridement did not occur for more than 1 week.

There is no antibiotic that can replace a proper, diligent surgical debridement.

Unfortunately, the initial apparently minor nature of the injury combined with the delay in progression to severe inflammation frequently results in a delayed referral. One study found that some patients will see up to seven physicians before receiving appropriate management [8]. Our study can confirm that this type of unfortunate scenario is still occurring. Furthermore, even though many members of the WJA carry a medical card alerting the medical staff of the potential injuries, in many cases, the patients are discharged home from the ED without even an observation period.

The management of such injuries consists of immediate exploration, extended as widely as is necessary with surgical debridement of all toxic material. Furthermore, areas of obvious necrosis should be excised, and the wound should be left open. Serial surgical debridement may well be necessary and should not be overlooked from the outset in the management scheme determined by the attending clinician(s). Open wound management has been shown to offer the best results for these injuries. One series reported a digit salvage rate of 84% and a return to normal hand function in 64% of patients, though amputation may still be necessary in certain cases.

The prognosis for the patient will be adversely affected by extreme contamination, massive blood transfusion, and suboptimal debridement before transfer or any delay in transfer.

In heavily contaminated wounds, debridement is often only achieved in several stages. The initial debridement is performed within the constraints of damage control surgery, with the first priority being to stop any bleeding (either externally or internally) and with the second goal being to limit contamination. Marginal debridement where all necrotic tissue is removed but where injured and potentially viable tissue is retained is an appropriate level of debridement during the first operation [5, 21].

At the second look stage, the need for further debridement must be evaluated. The wound should again be left open, or if all of the tissue is viable, a delayed primary closure or a reconstructive technique are suitable options to be considered. These decisions must be made by an experienced team and ideally in a trauma center.

Wound swabs, as well as tissue samples, should be taken as soon as possible and sent for microbiological and histological examination such that the presence of fungal spores can be identified [21]. If fungal contamination is suspected, samples should be taken of both healthy and dead muscle tissue.

High-pressure water jet injuries should always be considered surgical emergencies and treated as a severe trauma in a tertiary hospital whenever possible until demonstrated otherwise in the clinical environment. The management of such injuries should start from the very beginning, at the scene in controlling the hemorrhage, and across the second stage consisting of source hemorrhage control or emergency debridement. Open wound management has been shown to offer the best results for injuries. The antimicrobial treatment does not obviate the need for surgery.

## Conclusions

The risk of injury through hydraulic injection is common and present with respect to all types of hydraulic equipment and can also occur at relatively low pressure.

A review of the adverse incident reports, patient’s testimonies, and extant literature have identified that a lack of comprehension of the potential severity of injuries of this type is still the main obstacle to early and effective treatment. This appears largely due to the initial apparently minor nature of the presentation of an injury that can, unfortunately, have catastrophic consequences—such as amputation and death. With rapid, effective and informed treatment, there is thankfully a reduced risk of amputation or loss of function of the limb. The amputation risk is lower if wide surgical debridement occurs within 6 h of the injury. Due to the potential severity of infections and the range of pathogens early liaison with an infectious disease expert is essential.

From the fatalities reviewed, we conclude that the two main causes of death from HPWJI are severe TBI (or decapitations), all of which are fatal at the scene and massive traumatic hemorrhage (exsanguination) [22].

## Abbreviations

atm: Atmosphere
ED: Emergency department
IHPFII: Industrial high-pressure fluid injection injuries
HPWJI: High-pressure water jet injuries
mph: Miles per hour
NHS: National health service
psi: Pressure per square inch
TBI: Traumatic brain injury
UK: United Kingdom
WJA: The Water Jetting Association
